# Supplementary material for: Broad-complex transcription factor mediates opposing hormonal regulation of two phylogenetically distant arginine kinase genes in Tribolium castaneum
Source: Commun Biol. 2020 Oct 30;3:631. doi: 10.1038/s42003-020-01354-w (PMC7603314; doi:10.1038/s42003-020-01354-w)
Supplement: Supplementary file 1 — Supplementary Information [file 42003_2020_1354_MOESM1_ESM.pdf]

Supplementary information

Supplementary Figure 1: cDNA sequences and deduced amino acid sequences of TcAK1 and TcAK2

a

```
   1  ATTCTTCAGAGAGTGTGTTTCAAGCCAGACTTTCGACTAAAGAAAATCCACCATGGTTGACGCCGCAGTTTTGGAGAAACTGGAAGCCGG
                                                        M  V  D  A  A  V  L  E  K  L  E  A  G ·
  91  TTTCAAAAAACTGGAAGCTTCTGACTCCAAGTCCCTTTTGAAGAAATACTTGACCAGGGAGTTGTTCGACAAGCTGAAGACCAAGAAGAC
       F  K  K  L  E  A  S  D  S  K  S  L  L  K  K  Y  L  T  R  E  L  F  D  K  L  K  T  K  K  T ·
 181  TTCCTTCGGCTCCACTCTTCTCGATGTGATCCAGTCTGGTTTGGAAAACCACGATTCTGGAATTGGTATTTACGCCCCTGACGCTGACTC
       S  F  G  S  T  L  L  D  V  I  Q  S  G  L  E  N  H  D  S  G  I  G  I  Y  A  P  D  A  D  S ·
 271  CTATTCCGTGTTTGCCGACTTGTTCGACCCCATCATCGAAGACTACCATGGCGGTTTCAAGAAGACTGACAAGCACCCCCCCAAGGACTG
       Y  S  V  F  A  D  L  F  D  P  I  I  E  D  Y  H  G  G  F  K  K  T  D  K  H  P  P  K  D  W ·
 361  GGGCGATGTGAACGCCTTCGGCAACCTGGACCCTGCCGGCGAGTTCGTCGTGTCCACCCGTGTGCGCTGCGGCCGCTCCCTGGAGGGCTA
       G  D  V  N  A  F  G  N  L  D  P  A  G  E  F  V  V  S  T  R  V  R  C  G  R  S  L  E  G  Y ·
 451  CCCCTTCAACCCTTGCCTGACCGAGGAGCAGTACAAGGAGATGGAGCAGAAGGTGTCGTCGACTCTGTCGGGGCTCGAGGGCGAGCTCAA
       P  F  N  P  C  L  T  E  E  Q  Y  K  E  M  E  Q  K  V  S  S  T  L  S  G  L  E  G  E  L  K ·
 541  GGGCACCTTCTACCCCCTGACTGGCATGAGCAAGGAGGTGCAGCAGAAGCTGATCGACGACCACTTCCTGTTCAAGGAAGGCGACCGCTT
       G  T  F  Y  P  L  T  G  M  S  K  E  V  Q  Q  K  L  I  D  D  H  F  L  F  K  E  G  D  R  F ·
 631  CCTGCAGGCGGCCAACGCGTGCCGCTTCTGGCCCACCGGCCGTGGTATTTTCCACAACGACGCCAAGACCTTCCTGGTCTGGTGCAACGA
       L  Q  A  A  N  A  C  R  F  W  P  T  G  R  G  I  F  H  N  D  A  K  T  F  L  V  W  C  N  E ·
 721  AGAGGATCACTTGAGGATCATCTCCATGCAGATGGGGGGCGACCTGGGGCAGGTCTACCGTCGATTGGTGACGGGGGTCAACGATATCGA
       E  D  H  L  R  I  I  S  M  Q  M  G  G  D  L  G  Q  V  Y  R  R  L  V  T  G  V  N  D  I  E ·
 811  GAAGCGACTCCCCTTCTCGCACAGCGACAGGTTCGGGTTCCTCACTTTCTGCCCCACCAACCTTGGGACAACTGTTCGTGCCTCCGTTCA
       K  R  L  P  F  S  H  S  D  R  F  G  F  L  T  F  C  P  T  N  L  G  T  T  V  R  A  S  V  H ·
 901  CATCAAAGTTCCCAAACTGGCCGCCAACAAGGCCAAGTTGGACGAAGTCGCTGCCAAATTCAATCTGCAAGTGCGTGGAACTCGCGGCGA
       I  K  V  P  K  L  A  A  N  K  A  K  L  D  E  V  A  A  K  F  N  L  Q  V  R  G  T  R  G  E ·
 991  ACACACCGAGGCTGAGGGCGGCGTTTACGATATTTCCAACAAGAGACGCATGGGCCTCACTGAATTCGATGCTGTCAAGGAGATGTACGA
       H  T  E  A  E  G  G  V  Y  D  I  S  N  K  R  R  M  G  L  T  E  F  D  A  V  K  E  M  Y  D ·
1081  TGGCATTTCCGAAATCATCAAGATGGAAAAGGAGTTGTAAACCCCTTGCATCCGCAACATTCTAATCATACGTGATTCATCACTTATAAG
```

G I S E I I K M E K E L *

1171 CAATGACATAATTTTGAGTAAGTAAGTATATCTTGTTTGCCAAAGGTACCAGTGAAATGCTTATATTGCTAAGTTAATAAGATGGTGTCT

1261 GGACATGAGTTAGTGTATTCAGCTTTCTAGTCGACATTGTTTTCAAATGTGAAAAATTCTTACAAATTACAAAGCATTGGATTTTCCTTA

1351 TTTATTTATTTTAATGTATTTATGTAAAAACGAAAGTATTATTTTAAAAAGCAACTGCAAATTTATTACAACATTTAATAAAAGACGTCC

1441 AATATAAAAAAAAAAAAAAAAAAAAAAAAA

b

1 ATCAATTTTAAATGTCACAACACCTCAAATTATTGTATCCTTCTTTCATAAATATAATGGCAAAAGCAAAATGCCACAATTGCGAAACAA

M A K A K C H N C E T

91 AATGTGGCAAAGGCAAAATAAATCCCGAAGTTTTAACTAAATTAGAGGACGGCTTTCGGCGACTAGCAACTTCAAATTCCCAATCGCTCT

K C G K G K I N P E V L T K L E D G F R R L A T S N S Q S L

181 TAAAAAAATACTTGACCCAAGCTGTTTTCGACAAACTTAAAAGTAAGAAGACTGGTTTTGGCTCAACACTTCTGGACTGCATCCAGTCAG

L K K Y L T Q A V F D K L K S K K T G F G S T L L D C I Q S

271 GTCTCGAAAATCCAGACTCAAATATCGGTGTTTACGCACCTGACCCTGAAGCTTACACCGTTTTTGCCGAACTTTTTGACCCGATAATCG

G L E N P D S N I G V Y A P D P E A Y T V F A E L F D P I I

361 ACGAATACCATGGAGGGTTTAAGAAAACAGACCAACACCCTGCTAGCACTTGGGGGGACTTGAAGTCGATCGGAGACGTGGATCCTGATC

D E Y H G G F K K T D Q H P A S T W G D L K S I G D V D P D

451 AAAAATACGTTGTTTCGACCAGGATCAGGTGTGGACGCTCTTTGGCGGGTTATCCCTTCAACCCTTGCTTGACTGAAGCTCAGTACAAGG

Q K Y V V S T R I R C G R S L A G Y P F N P C L T E A Q Y K

541 AGATGGAGAAGAAGGTGTCGGGGACTTTGATGTCCCTAACCGGGGAACTTAAGGGAAAATATTACCCTTTGACGGGGATGTCAAAAAGGG

E M E K K V S G T L M S L T G E L K G K Y Y P L T G M S K R

631 ATCAGCAGCAGTTAATCGACGACCATTTCCTGTTTAAGGAAGGTGATAGGTTTTTACAAGCGGCCAATGCTTGTCGGTTCTGGCCCACAG

D Q QQ L I D D H F L F K E G D R F L Q A A N A C R F W P T

721 GCCGTGGCATTTTCCATAATACGAAAAAAACGTTCCTGGTTTGGCTCAATGAGGAGGATCACCTGAGGATTATCTCCATGCAAAAGGGTG

G R G I F H N T K K T F L V W L N E E D H L R I I S M Q K G

811 GAAACGTAGGGGAGGTCTACCAACGTTTGGTTTGTGCTGTTGAGGAGATTGAAACCAAGTTGAAGTTTTCGCGGAGTGATCGGTTTGGTT

G N V G E V Y Q R L V C A V E E I E T K L K F S R S D R F G

901 ACTTGACTTTTTGCCCGACCAATTTAGGTACAACGATAAGAGCGTCAGTGCATATTCAAGTGCCCAAGTTGGCCAAAGATAAGGCAAAAT

Y L T F C P T N L G T T I R A S V H I Q V P K L A K D K A K

991 TGGAAGCTGTAGCAAATCAGTTTAATTTGCAAGTGAGGGGGACTAGAGGAGAGCATAGTGAAGCTGAGGAAGGTGTTTATGATATTTCGA

L E A V A N Q F N L Q V R G T R G E H S E A E E G V Y D I S

1081 ATAGGAGGAGATTAGGACTTACGGAGTATGACGCCGTCAAAGAGATGCATGATGGTATTTTGAAAATTATTGAAATGGAGAGTTGCATGT

N R RR L G L T E Y D A V K E M H D G I L K I I E M E S C M

1171 GAATGGGAAAGTTGCAGCAAAGGAATAACAAATTTTCATGTTTTTTTTGAGAATTGTTTGTTGTTTTTAGTAAAAATTCAAAAGCAGAAA

*

1261 AAAAAAAAAAAAAAAAAAAAAAA

a cDNA sequences and deduced amino acid sequences of TcAK1. b cDNA sequences and deduced amino acid sequences of TcAK2 in *Tribolium castaneum*. The stop codon is indicated by an asterisk (*). The potential polyadenylation signal AATAAA are shaded**.**

Supplementary Figure 2: Phylogenetic relationships of the arginine kinase proteins

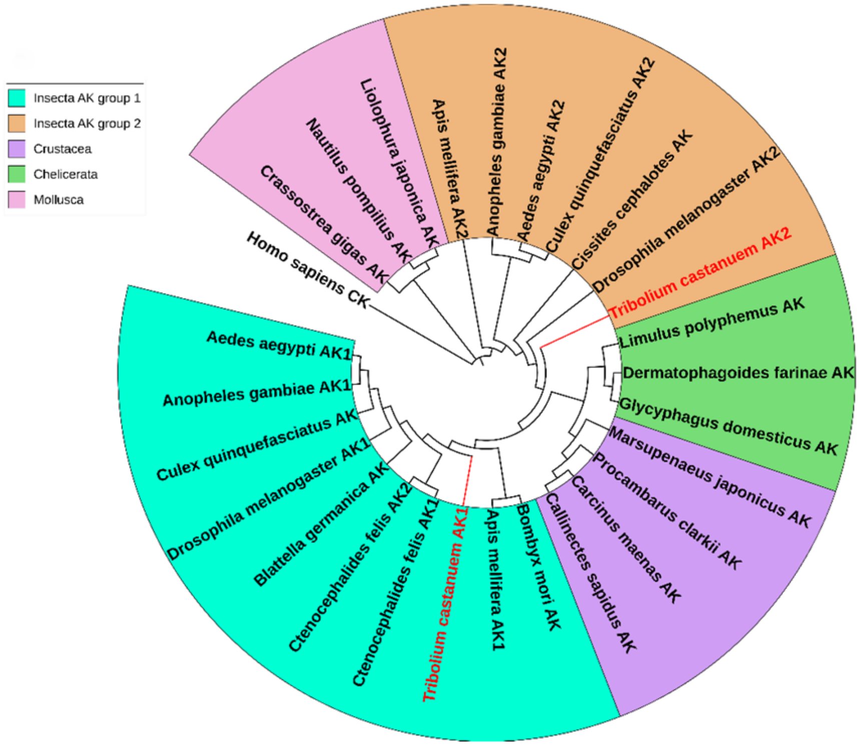

Phylogenetic relationships of the AK proteins from *Tribolium castaneum* and other species. The Neighbor-joining tree was generated in MEGA5 with 1000 bootstrapping. AK sequences are obtained from the following GenBank entries: BAD11950 for *Crassostrea gigas* AK; BAA95594 for *Nautilus pompilius* AK; O15990 for *Liolophura japonica* AK; XP_026301800 for *Apis mellifera* AK2; XP_321020 for *Anopheles gambiae* AK2; XP_001657389 for *Aedes aegypti AK*2; XP_001849654 for *Culex quinquefasciatus* AK2; BAF49173 for *Cissites cephalotes* AK;

AAS93705 for *Drosophila melanogaster* AK2; NP_001301013 for *Limulus polyphemus* AK; AAP57094 for *Dermatophagoides farinae* AK; ABU97471 for *Glycyphagus domesticus* AK; AID47194 for *Marsupenaeus japonicus* AK; AFA45339 for *Procambarus clarkii* AK; Q9U9J4 for *Carcinus maenas* AK; Q9NH49 for *Callinectes sapidus* AK; XP_021202112 for *Bombyx mori* AK; NP_001011603 for *A. mellifera* AK1; XP_026473221 for *Ctenocephalides felis* AK1; XP_026474404 for *C. felis* AK2; AEV23883 for *Blattella germanica* AK; NP_729446 for *D. melanogaster* AK1; XP_001849654 for *C. quinquefasciatus* AK; XP_001688698 for *Anopheles gambiae* AK1; XP_021697332 for *A. aegypti* AK1. *Homo sapiens* creatine kinase (NP_001015001) was used as the outgroup.

Supplementary Figure 3: Amino acid sequence alignments.

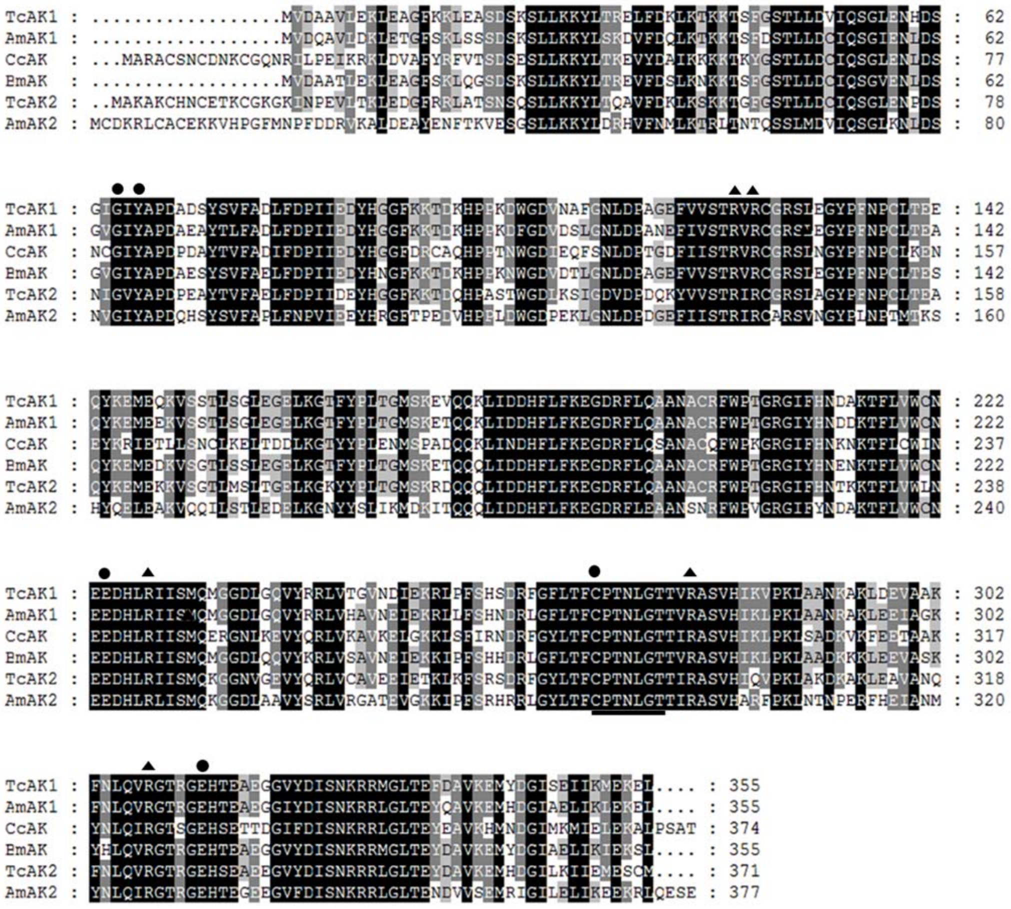

Amino acid sequence alignments of TcAK1 and TcAK2 with AmAK1 and AmAK2 in *Apis mellifera*, BmAK in *Bombyx mori*, and CcAK in *Cissites cephalotes*. Identical amino acids are shown in black boxes and similar amino acids are highlighted in gray boxes. Gaps have been

introduced to permit alignment. GenBank accession numbers are shown in Supplementary **Figure** 2. The phosphagen kinase signature sequence CP(S/T)N(I/L)GT is underlined. The key residues involved in the binding guanidino substrate are marked by filled circles. The five conserved arginine residues involved in ATP binding are highlighted by black triangles.

Supplementary Figure 4: Genomic structure of *TcAK1* and *TcAK2*.

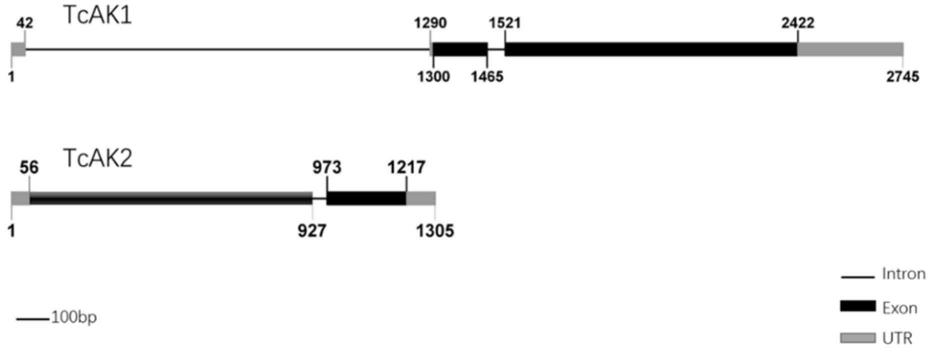

The genomic structure of *TcAK1* and *TcAK2* highlighting the intron/exon organization of the two genes.

Supplementary Figure 5: Detection of TcAK1 and TcAK2 protein expression.

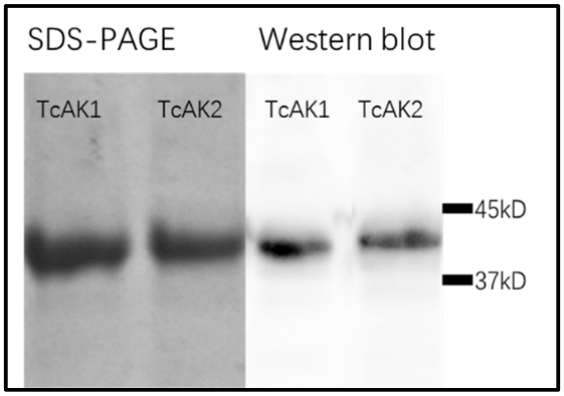

10% SDS-polyacrylamide gel electrophoresis (SDS-PAGE) and western blot analysis of recombinant TcAK1 and TcAK2 expressed in *Escherichia coli*. The relative positions of protein size markers are indicated in kDa.

Supplementary Figure 6: Guanidino substrate specificity of 6×His-TcAK1 and 6×His-TcAK2 in the direction of phosphagen synthesis.

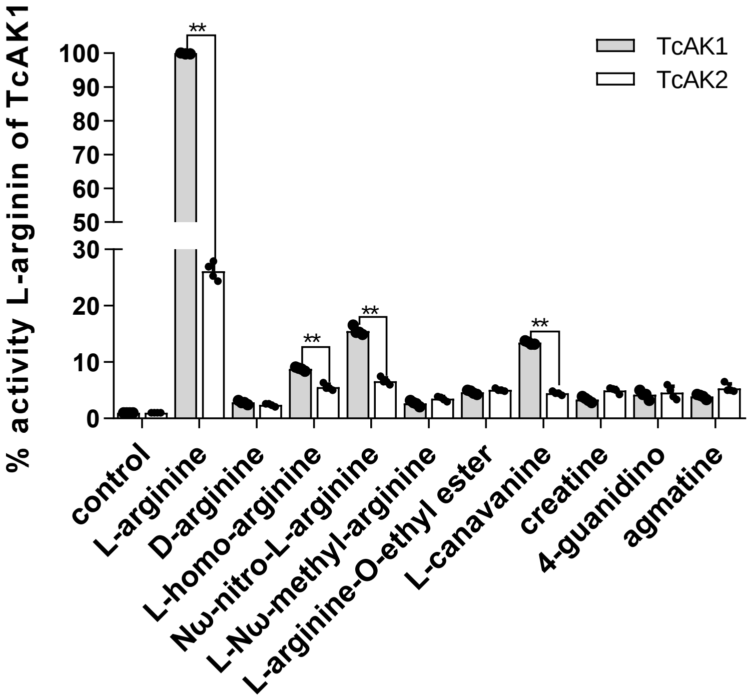

Standard arginine kinase conditions were applied and the reaction rates at 5 mM guanidino substrate concentrations were determined. Data are expressed as mean ± S.E.M (n = 3 biologically independent replicates). Asterisks indicate differences statistically significant at $^{**}P < 0.01$ (student's t-test).

Supplementary Figure 7: Knockdown efficiency of *TcAK1* and *TcAK2* expression.

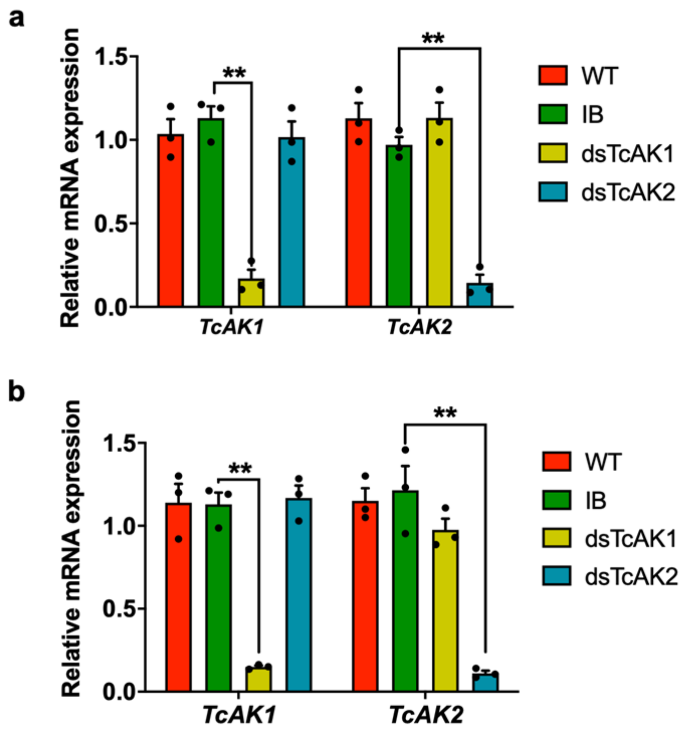

a Knockdown efficiency of TcAK1 and TcAK2 expression after injection of dsRNA into 20-day-old larvae. **b** Knockdown efficiency of TcAK1 and TcAK2 expression after injection of dsRNA into 2-day-old pupae. RT-qPCR was used to quantify mRNA levels of dsRNA on the fourth day after dsRNA injection in larvae or on the fourth day after adult emergence after dsRNA injected. WT: the uninjected wild-type group, IB: the buffer-injected group, dsTcAK1, dsTcAK2: the dsRNA injected group. *Tribolium* ribosomal protein 3 (rps3) transcript with the same cDNA template served as an internal control. Data are expressed as mean ± S.E.M (n = 3 biologically independent replicates). Asterisks indicate statistically significant differences from control groups (student's t-test, $P < 0.01$).

Supplementary Figure 8: Stress-induced mRNA and protein expressions of TcAK1 and TcAK2.

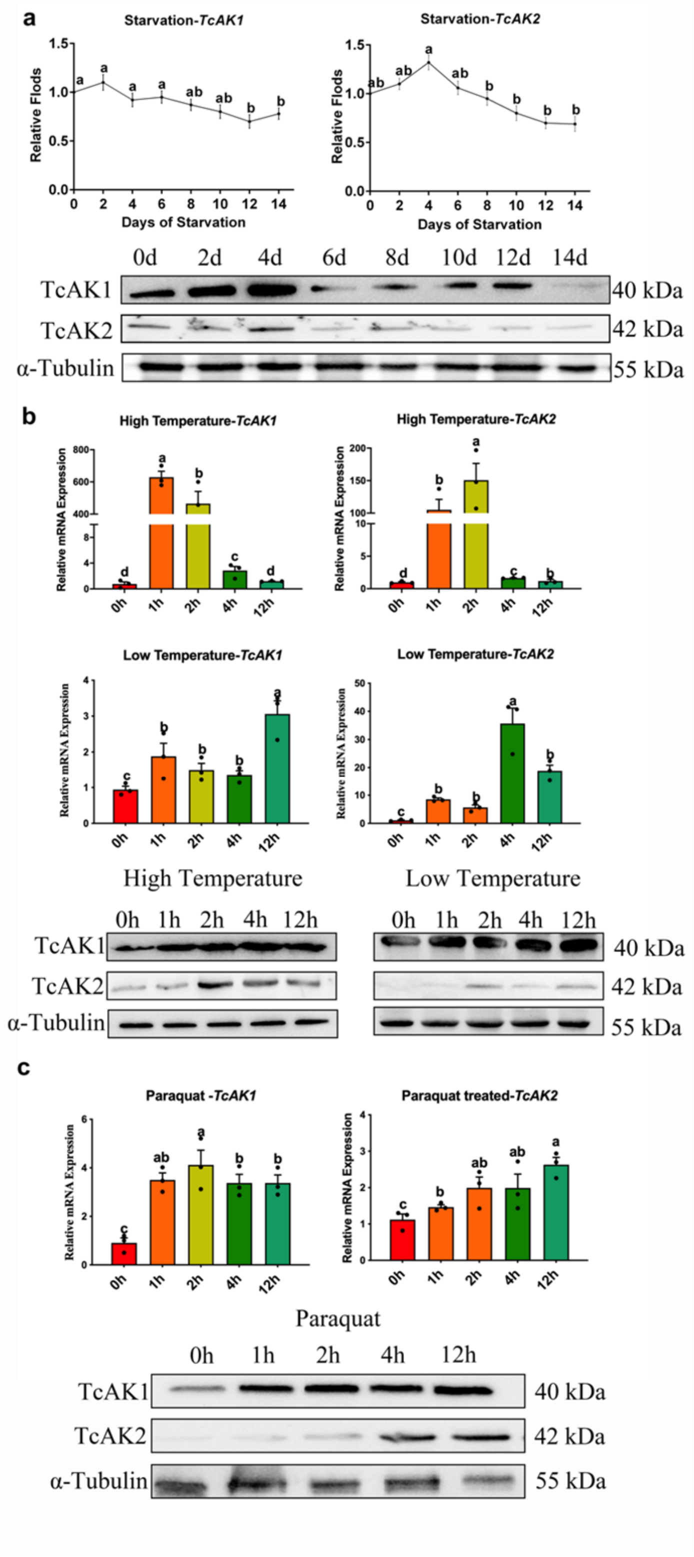

a Stress-induced mRNA and protein expressions of TcAK1 and TcAK2 under treatments of starvation. **b** Stress-induced mRNA and protein expressions of TcAK1 and TcAK2 under treatments of 45°C, 4°C. **c** Stress-induced mRNA and protein expressions of TcAK1 and TcAK2 under treatments of paraquat. mRNA expression was determined by qPCR. Data are expressed as mean ± S.E.M (n = 3 biologically independent replicates). Histogram bars annotated with the same lowercase letters are not significantly different (one-way ANOVA, $P < 0.05$). Protein expression was determined by western blot and α-Tubulin was used as a loading control. These experiments were repeated at least three times with similar results.

Supplementary Figure 9. The gray value ratio of TcAK1 and TcAK2 to α-tubulin in each treatment.

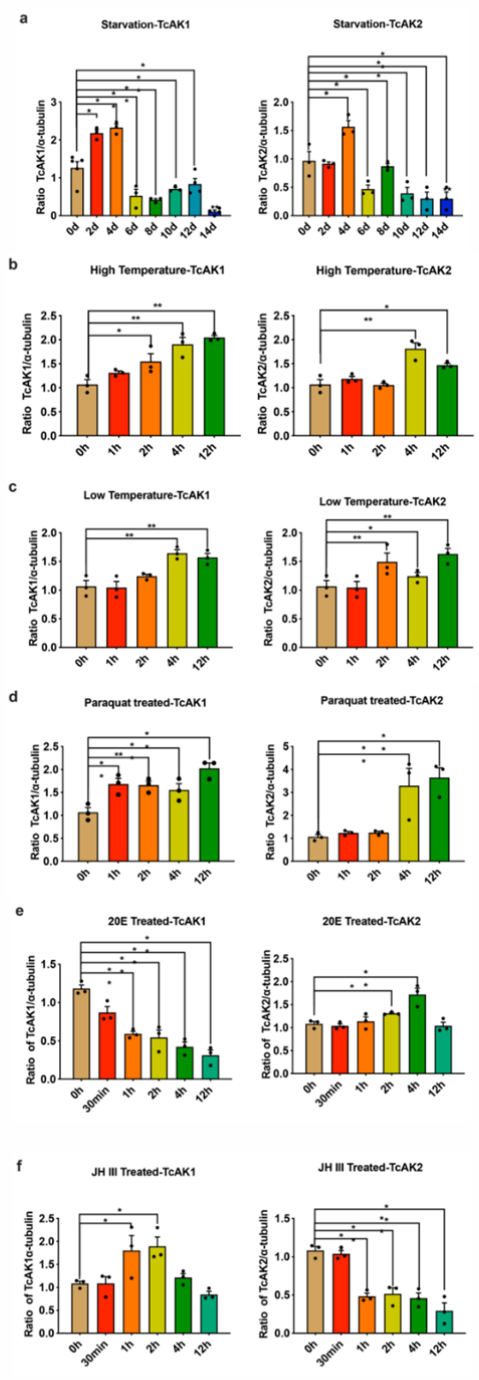

a The relative expressions of protein under treatments of starvation. **b** The relative expressions of protein under treatments of high temperature 45°C. **c** The relative expressions of protein under treatments of low temperature 4°C. **d** The relative expressions of protein under treatments of paraquat. **e** The relative expressions of protein under treatments of 20E. **f** The

relative expressions of protein under treatments of JH III. The gray value ratio of TcAKs to α-tubulin in each group was measured. The ratio in the 0 h group was set as a reference, and all experimental ratios were compared with the 0 h group. These experiments were repeated at least three times with similar results. Data are expressed as mean ± S.E.M (n = 3 biologically independent replicates). Asterisk indicates differences statistically significant at ∗$P < 0.05$ and ∗∗$P < 0.01$ (student's t-test).

Supplementary Figure 10: The effects of knockdown of expression of *TcAK1* and *TcAK2* on the tolerance to stress.

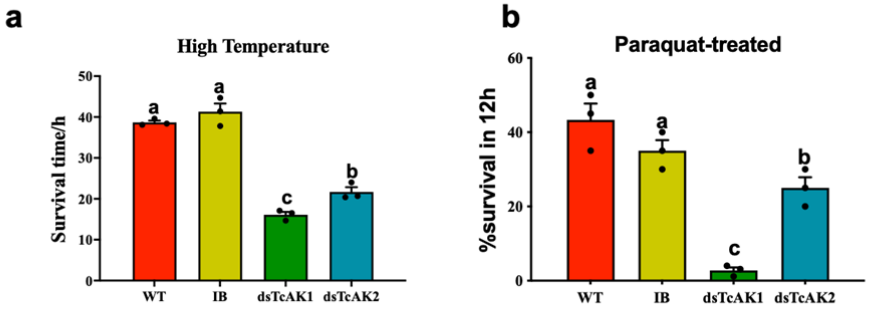

a The effects of knockdown of expression of *TcAK1* and *TcAK2* on the tolerance to high temperature (45°C). **b** The effects of knockdown of expression of *TcAK1* and *TcAK2* on the tolerance to paraquat. Data are expressed as mean ± S.E.M (n = 3 biologically independent replicates). Histogram bars annotated with the same lowercase letters are not significantly different (one-way ANOVA, $P < 0.05$).

Supplementary Figure 11: The effects of hormonal treatment on ATP content *in vivo*.

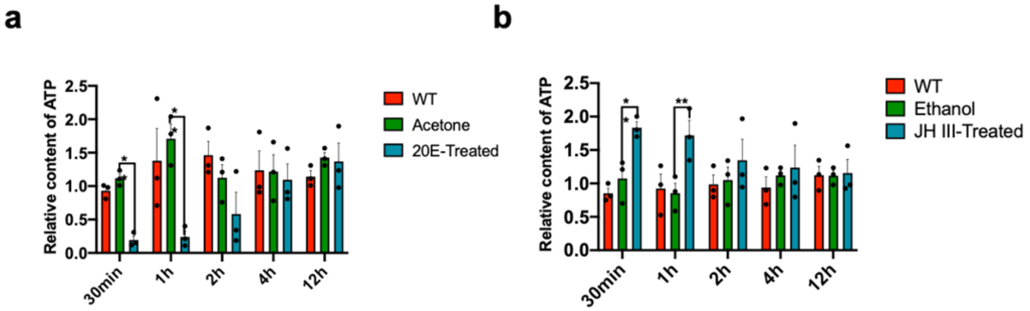

a The effects of 20E treatment on ATP content *in vivo*. **b** The effects of JH III treatment on ATP content *in vivo*. Data are expressed as mean ± S.E.M (n = 3 biologically independent replicates). Asterisk indicates differences statistically significant at ∗∗P< 0.01 (student's t-test).

Supplementary Figure 12: Specificity of antibodies against TcAK1 and TcAK2.

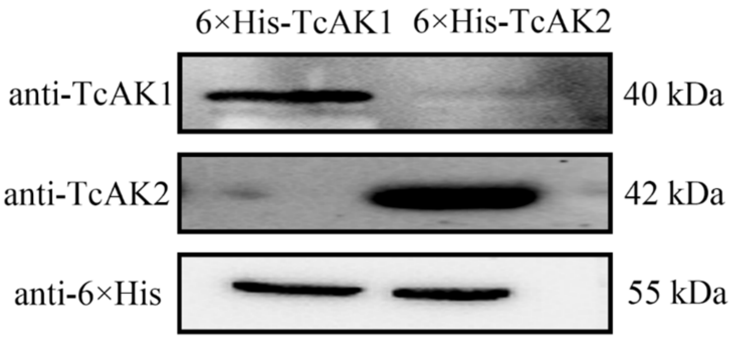

Two identical sets of samples, each containing about 100 ng of recombinant bacterially expressed 6×His-TcAK1 and 6×His-TcAK2, were resolved on SDS-PAGE (15% polyacrylamide) and transferred to PVDF. Each half of the membrane containing one set of samples was separately blotted with either TcAK1 or TcAK2 antibodies. Simultaneously, incubating with anti His-tag antibody was used as the control. All experiments were repeated three times with similar results.

Supplementary Figure 13: Detection of TcBR-C-Z2 protein expression

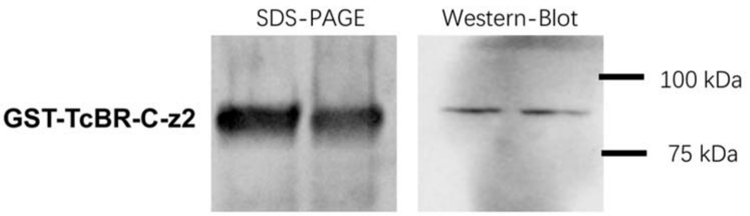

10% SDS-polyacrylamide gel electrophoresis (SDS-PAGE) and western blot analysis of recombinant TcBR-C-z2 expressed in *Escherichia coli* with anti-GST antibody. The relative positions of protein size markers are indicated in kDa.

Supplementary **Figure** 14: Uncropped blots for Figures and Supplementary Figures.

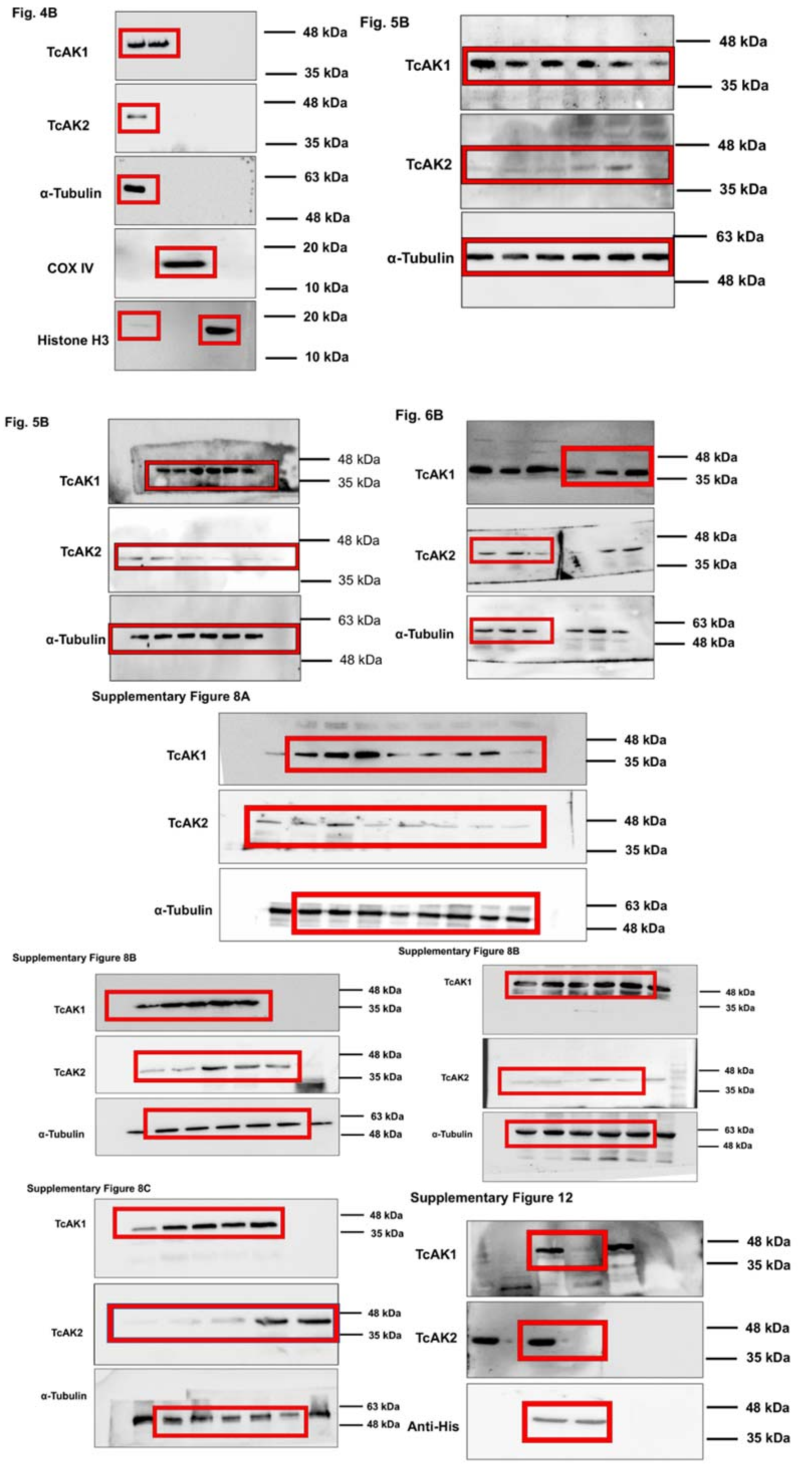

Enclosed blot areas are used for corresponding figure parts

Supplementary Table 1. Oligonucleotide primers used for RT-PCR, RACE, qPCR, dsRNA synthesis and vector construction [a]

Primer name	Sequence (5' to 3')	Description
TcAK1F1	ATGGTTGACGCCGCAGTTTT	TcAK1 RT-PCR
TcAK1R1	CAAGGGGTTTACAACTCCTT	
TcAK2F1	ATGGCAAAAGCTAAATGCCA	TcAK2 RT-PCR
TcAK2R1	TCACATGCAACTCTCCATTT	
TcAK1R2	TACTGCTCCTCGGTCAGGCAAGGGT	TcAK1 5′-RACE
TcAK1R3	ACAAGTCGGCAAACACGGAATAGGA	
TcAK1F2	GCGACAGGTTCGGGTTCCTCACTTT	TcAK1 3′-RACE
TcAK1F3	CTGCCAAATTCAATCTGCAAGTGCG	
TcAK2R2	CAGTCAAGCAAGGGTTGAAGGGATA	TcAK25′-RACE
TcAK2R3	TTGCTAGTCGCCGAAAGCCGTCCTC	
TcAK2F2	AGGAGGATCACCTGAGGATTATCTC	TcAK2 3′-RACE
TcAK2F3	CCCGACCAATTTAGGTACAACGATA	
TcAK1F4	ACTACCATGGCGGTTTCAAG	TcAK1 RT-qPCR
TcAK1R4	CTCCTTGTACTGCTCCTCGG	
TcAK2F4	AAAACAGACCAACACCCTGC	TcAK2 RT-qPCR
TcAK2R4	AAAGTCCCCGACACCTTCTT	
Tcrps3F1	ACCGTCGTATTCGTGAATTGAC	rps3 RT-qPCR
Tcrps3R1	ACCTCGATACACCATAGCAAGC	
TcAK1F5	GGGGTACCATGGTTGACGCCGCAGTTTT	TcAK1 ORF
TcAK1R5	CCCCCGGGTTACAACTCCTTTTCCATCT	
TcAK2F5	GGGGTACCATGGCAAAAGCAAAATGCCA	TcAK2 ORF
TcAK2R5	CCCCCGGGTCACATGCAACTCTCCATTT	
AK1RiF	ACAGCGACAGGTTCGGGTTC	TcAK1 dsRNA
AK1RiR	TTTCGGAAATGCCATCGTAC	

AK2RiF	ATCGGTTTGGTTACTTGACT	TcAK2 dsRNA
AK2RiR	CTTTGACGGCATCATACTCC	
TcAK1-promoterF	ctatcgataggtaccgagctcGTACAACGAACTAAAAAAGTAAAATAACTAACA	Promoter of TcAK1
TcAK1-promoterR	gctagcttgatgccattcatgTGCTAGAGATTAAGGGCTCAC	
TcAK2-promoterF	ctatcgataggtaccgagctcACAGAGACAATTAAATAAATAAAATATTTTGAT	Promoter of TcAK1
TcAK2-promoterR	gctagcttgatgccattcatgTATATTTATGAAAGAAGGATACAATAATTTGA	
pAC-TcBR-C-F	cgagctgtacaagccaagcttgATGGTAGATACACAACACTTCTGTCTGC	
pAC-TcBR-C-z1-R	gccctctagactcgagcggccgcTTAGAGATAGAACGAATGCGGCG	TcBR-C-z1 ORF
pAC-TcBR-C-z2-R	gccctctagactcgagcggccgcCTAAAAGAACTTTATGTCGATGTCCC	TcBR-C-z2 ORF
pAC-TcBR-C-z3-R	gccctctagactcgagcggccgcTCACTGGAGGCGCGGCTG	TcBR-C-z3 ORF
pAC-TcBR-C-z4-R	gccctctagactcgagcggccgcTTATTTTTGTCTGCGATGGTAAATACT	TcBR-C-z4 ORF
pAC-TcBR-C-z5-R	gccctctagactcgagcggccgcGATGAAGCAGTCATTCTCAAATTTG	TcBR-C-z5 ORF
TcShadeF1[b]	CCGGCCTACAAAACACTCAT	Tcshade dsRNA
TcShadeR1[b]	CAATCCCAAAGTGACACACG	
TcShadeF2[b]	TCGTACTCGAAAGCGTGCGTTACT	Tcshade RT-qPCR
TcShadeR2[b]	GCTCCGGTTTGAACTTTGAAGCGT	
TcPhantomF1[b]	TGTCATCCAAGCGTTTCTTG	Tcphantom dsRNA
TcPhantomR1[b]	AGCAATTTCCAGCTCTTCCA	
TcPhantomF2[b]	TGATCTCCGACGCCAAACTCATCA	Tcphantom RT-qPCR

TcPhantomR2 [b]	GCAAATCAGGCCGAAACCCTTCAT	
TcJHAMTF1 [b]	ATGAACAAAGCCTCACTGTACTCAA	TcJHAMT dsRNA
TcJHAMTR1 [b]	CTCTGTTCCACCACCCAATGCAA	
TcJHAMTF2 [b]	CATCTCGCCCTATCACCATTCG	TcJHAMTRT-qPCR
TcJHAMTR2 [b]	CCGCTGAAACCGATTTTGACAA	
TcKr-h1F1[c]	GAACGAATCCTCCTGCTCATCCA	TcKr-h1dsRNA
TcKr-h1R1 [c]	CTTGGCGGAAGACTCAACTCCC	
TcKr-h1F2 [c]	CCAGCGGTCTCTTTCGTCGTTT	TcKr-h1RT-qPCR
Tc Kr-hR2 [c]	CGTCCTCCACTAACTTCTCGGTGAA	
TcBR-Ci-F[d]	TCGGCAACAACAACAATAAC	TcBR-C core region dsRNA
TcBR-Ci-R[d]	CATCGGT TCGCTCTTCAC	
TcBR-C-F[d]	CACAACACTTCTGTCTGCGGTG	TcBR-C core region qPCR
TcBR-C-R[d]	CACAGGGTGTTTGCAAGGAG	
pAC-DmB-Rc-F	cgagctgtacaagccaagcttgATGGACGACACACAGCACTTCTG	
pAC-DmBRc-z1-R	gccctctagactcgagcggccgcCTATGAAGAGGAGTGGTGATTGAGC	DmBR-C-z1 ORF
pAC-DmBRc-z2-R	gccctctagactcgagcggccgcCTAGTTGCTCGTGGTGGCAAT	DmBR-C-z2 ORF
pAC-DmBRc-z3-R	gccctctagactcgagcggccgcTCAGATCGACGAGTTGAACTGG	DmBR-C-z3 ORF
pAC-DmBRc-z4-R	gccctctagactcgagcggccgcTTATAAGAAGTCCATGCACGGTTT	DmBR-C-z4 ORF
GST-TcBRc-z2F	gatctggttccgcgtggatccATGGTAGATACACAACACTTCTGTCTGC	
GST-TcBRc-z2R	tcagtcagtcacgatgcggccgcCTAAAAGAACTTTATGTCGATGTCCC	GST fusion protein expression
DmRp49-F[e]	GACAGTATCTGATGCCCAACA	Rp49 RT-qPCR
DmRp49-R[e]	CTTCTTGGAGGAGACGCCGT	
DmAK1-qF	CTCGATATTGGCCAACGGGA	DmAK1 RT-qPCR
DmAK1-qR	AGCCAAGTCGTTCATCCCTG	

DmAK2-qF	GATGCCGAGGCTTACACAGT	DmAK2 RT-qPCR
DmAK2-qR	ATCACGTACTCGTTGGTGGG	

[a]The restriction enzyme sites introduced by the primer as well as the additional 15 nucleotides complementary to the cloning site on the plasmid were underlined.

[b] All these primers were derived from the following reference: Parthasarathy, R., Sun, Z., Bai, H. & Palli, S.R. Juvenile hormone regulation of vitellogenin synthesis in the red flour beetle, *Tribolium castaneum*. *Insect Biochem Mol. Biol.* **40,** 405-414 (2010).

[c] All these primers were derived from the following reference: Minakuchi, C., Namiki, T. & Shinoda, T. Krüppel homolog 1, an early juvenile hormone-response gene downstream of Methoprene-tolerant, mediates its anti-metamorphic action in the red flour beetle *Tribolium castaneum*. *Dev Biol.* **325,** 341-350 (2009).

[d]All these primers were derived from the following reference: Suzuki, Y., Truman, J. W. & Riddiford, L. M. The role of broad in the development of *Tribolium castaneum*: implications for the evolution of the holometabolous insect pupa. *Development* **135,** 569-577 (2008).

[e]All these primers were derived from the following reference: Jia, Q. et al. Juvenile hormone and 20-hydroxyecdysone coordinately control the developmental timing of matrix metalloproteinase–induced fat body cell dissociation. *J. Biol. Chem.* **292,** 21504-21516 (2017)

Supplementary Table 2. The Br-C binding sites in the promoter regions of TcAKs predicted by JASPAR (http://jaspar.genereg.net/) and TFBIND (http://tfbind.hgc.jp/)

Gene	Detailed Matrix Information	position		Strand	Sequence
		From	To		
**TcAK1**	Broad-Complex Z1 Zinc Finger isoform	-1002	-989	-	tttttgtcaatcac
	Broad-Complex Z2 Zinc Finger isoform	-269	-262	-	aaatagat
		-601	-594	-	aaataata
		-650	-643	-	taatagta
		-1468	-1461	-	aaataggt
		-1857	-1850	-	aaataaaa
		-2141	-2134	+	ttttattt
	Broad-Complex Z3 Zinc Finger isoform	-1726	-1716	-	taaacaatagg
	Broad-Complex Z4 Zinc Finger isoform	-1913	-1903	-	tttttttataa
		-2160	-2150	+	taataaaatat
**TcAK2**	Broad-Complex Z1 Zinc Finger isoform	-1312	-1299	+	ataattaaaaaaaa
	Broad-Complex Z2 Zinc Finger isoform	-279	-272	-	aaatagaa
		-610	-603	-	aaatagtt
		-717	-710	+	tgctattt
		-1104	-1097	+	tattattt
		-1298	-1291	+	tattattt
		-1680	-1673	+	acctattt
		-1933	-1926	+	aaataaaa
		-2209	-2202	+	aattagta
		-2067	-2060	-	ttctattg
	Broad-Complex Z3 Zinc Finger isoform	-535	-525		aaattagtttt
		-1817	-1807	-	gtttttgtttt

Broad-Complex Z4 Zinc Finger isoform	-859	-849	+	ttgtaaaaaaa
	-906	-896	+	ttataaacgaa
	-1020	-1010	-	ttggtttacta
